# Comparison of results obtained using clot-fibrinolysis waveform analysis and global fibrinolysis capacity assay with rotational thromboelastography

**DOI:** 10.1038/s41598-024-58436-6

**Published:** 2024-03-31

**Authors:** Takumi Tsuchida, Mineji Hayakawa, Osamu Kumano

**Affiliations:** 1 Division of Acute and Critical Care Medicine, Department of Anesthesiology and Critical Care Medicine, Faculty of Medicine, Hokkaido University, Sapporo, Japan; 2 Sysmex Corporation, Kobe, Japan; 3 Health and Medical Research Institute, National Institute of Advanced Industrial Science and Technology (AIST), Takamatsu, Japan

**Keywords:** Global fibrinolysis assays, Tissue-type plasminogen activator, Clot-fibrinolysis waveform analysis, Global fibrinolysis capacity assay, Rotational thromboelastography, Hyperfibrinolysis status, Biomarkers, Medical research

## Abstract

Global fibrinolysis assays detect the fibrinolysis time of clot dissolution using tissue-type plasminogen activator (tPA). Two such assays, clot-fibrinolysis waveform analysis (CFWA) and global fibrinolysis capacity (GFC) assay, were recently developed. These were compared with rotational thromboelastography (ROTEM). Healthy donor blood samples were divided into four groups based on tPA-spiked concentrations: 0, 100, 500, and 1000 ng/mL. CFWA and GFC fibrinolysis times, including 4.1 µg/mL and 100 ng/mL tPA in the assays, were determined, denoted as CFWA-Lys and GFC-Lys, respectively. Statistical differences were recognized between tPA concentrations of 0 and 500/1000 ng/mL for CFWA-Lys, and 0 and 100/500/1000 ng/mL for GFC-Lys. The correlation coefficients with lysis onset time (LOT) of extrinsic pathway evaluation and intrinsic pathway evaluation in ROTEM were statistically significant at 0.610 and 0.590 for CFWA-Lys, and 0.939 and 0.928 for GFC-Lys, respectively (*p*-values < 0.0001 for all correlations). Both assays showed significant correlations with ROTEM; however, the GFC assay proved to have better agreement with ROTEM compared with the CFWA assay. These assays have the potential to reflect a hyperfibrinolysis status with high tPA concentrations.

## Introduction

The balance between the pro-coagulant, anti-coagulant, and fibrinolytic systems is important and must be well controlled to maintain normal hemostasis in the circulating blood. When this relationship is unbalanced, hemorrhage or thrombosis may appear as the clinical manifestations. The coagulation and fibrinolysis systems are remarkably altered in patients in critical care situations. For example, patients with severe trauma experience hemorrhage associated with hyperfibrinolysis in the early trauma period, followed by thrombus formation associated with hypercoagulability activation in the subacute period^1^. In patients with sepsis, the coagulation reaction is activated, but fibrinolysis is suppressed, which induces organ dysfunction because blood circulation in the organs is obstructed by the microthrombi generated by the coagulation reaction^2^. This induces septic disseminated intravascular coagulation (DIC), and the diagnosis of DIC in the early phase of sepsis is critical for improving a patient’s condition^3^. Therefore, it is important to assess both the coagulation and fibrinolysis status to fully understand a patient’s condition, especially in critically ill patients.

Several biomarkers, including fibrinogen, fibrin/fibrinogen degradation products, D-dimers, and thrombin-antithrombin complex, are used to identify the coagulation and fibrinolysis status in critical care situations^4,5^; however, it is difficult to understand accurately the coagulation and fibrinolysis status via the interpretation of the results of several biomarkers. Therefore, a global assay that reflects both the coagulation and fibrinolytic reactions in a single measurement is desirable. Viscoelastic testing, including thromboelastography and thromboelastometry, is a classical global assay that is used to measure the global viscoelastic properties of whole-blood clot formation under shear stress. This assay provides detailed information about clotting kinetics from clot formation to degradation. Rotational thromboelastometry (ROTEM) is a point-of-care device for this test, which has been widely used to detect hypo- as well as hypercoagulable conditions^6–12^. Additionally, two kinds of global fibrinolysis assays, including clot-fibrinolysis waveform analysis (CFWA) and global fibrinolysis capacity (GFC) assay, were developed recently. CFWA evaluates the fibrinolysis time from clot formation to dissolution using an automated coagulation analyzer. In this assay, plasma samples are mixed with activated partial thromboplastin time (APTT) reagent and incubated with CaCl_2_ solution containing 4.1 µg/mL of tissue-type plasminogen activator (tPA), followed by detection of light transmittance by the instrument^2^. Several studies have shown the usefulness of this assay for the assessment of patients using direct oral anticoagulants, patients with COVID-19, and critically ill patients^13–15^. In the GFC assay, plasma samples are activated with silica with 100 ng/mL tPA, followed by incubation with thrombin and CaCl_2_ solution. The change in light transmittance is monitored, and the fibrinolysis time is measured using a Lysis Timer instrument developed by HYPHEN BioMed (Neuville-sur-Oise, France)^3^. The correlation between the fibrinolysis time and the related fibrinolysis markers of plasminogen activator inhibitor-1, thrombin activatable fibrinolysis inhibitor, and tPA has been reported, and it was shown that GFC assay reflected the fibrinolysis status^16^. 

Recently, these coagulation and fibrinolysis global assays have been used to understand the fibrinolysis status because of the potential, especially in patients with COVID-19 which enhances coagulation and fibrinolysis reactions and disturbs the balance, and their usefulness has been assessed in some studies^14,17,18.^ However, the interpretation of results and data obtained using different methods in the three assays of ROTEM, CFWA, and GFC are not comparable because the activators and tPA concentrations are different, and these factors can substantially affect the fibrinolysis time. In our previous study, we showed that the tPA concentration used in CFWA was higher than that under physiological conditions and that the assay reflected plasminogen and a_2_-plasmin inhibitor levels^15^. The tPA concentration in the GFC assay was lower and the fibrinolysis time was longer than that in CFWA^16^. Although the tPA concentration is an important factor that can affect the assay characteristics, no study has compared these assays to date. Furthermore, the number of studies comparing these two assays with ROTEM, a classical coagulation fibrinolysis global assay, is limited. In the present study, the difference between the CFWA and GFC assays was investigated and compared with ROTEM with respect to the tPA concentration in normal donor plasma samples spiked with tPA. We focused on tPA concentrations because the tPA concentrations are different among the assays. Additionally, the characteristics of the parameters of these assays were compared with those of ROTEM in fibrinolysis-enhanced samples for validation.

## Materials and methods

### ROTEM method

**Figure 1:**
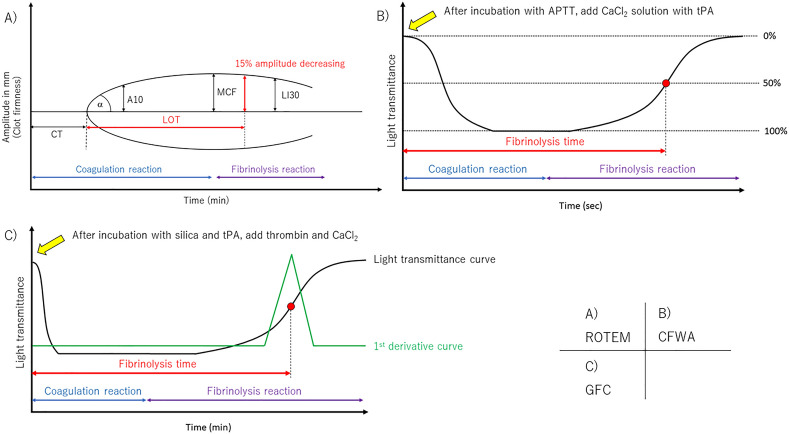
Overview of the parameters in the ROTEM, CFWA, and GFC assays. (**A**) ROTEM has several coagulation and fibrinolysis parameters. The CT is the time in minutes from the start to reach an amplitude of 2 mm. LOT is the time from CT to the point of a 15% decrease in the amplitude relative to the MCF. (**B**) CFWA is an assay that uses APTT reagent and CaCl_2_ solution containing tPA. The fibrinolysis time is detected as the time from the start to 50% light transmittance change in the fibrinolysis reaction. (**C**) In the GFC assay, the light transmittance curve and the first derivative curve were obtained in a single measurement, and the fibrinolysis time was defined as the time from the start to the point with the maximum value in the first derivative curve.

Whole blood samples were analyzed using EXTEM and INTEM for extrinsic and intrinsic coagulation pathway evaluation in ROTEM Delta (IL Japan, Tokyo, Japan), respectively, according to the manufacturer’s instructions. Coagulation and fibrinolysis reactions were monitored by measuring the viscoelasticity of the blood in the instrument. An overview of each parameter in the ROTEM is shown in Fig. 1a. Clotting time (CT) was defined as the time in minutes to reach an amplitude of 2 mm. Angle (a) was determined by creating a tangent line from the point of clot initiation to the slope of the developing curve. Maximum clot firmness (MCF) was the peak amplitude indicating clot strength. Lysis onset time (LOT) was employed as the fibrinolysis parameter in this study, and it was calculated as the time from CT to the initiation of clot lysis and defined as a 15% decrease in amplitude relative to the MCF. Lysis index after 30 min (LI30) was defined as the amplitude measured 30 min after CT/MCF.

### CFWA method

The detailed CFWA method has been described elsewhere^2^. Briefly, alteplase (Kyowa Kirin, Tokyo, Japan), which is recombinant tPA (r-tPA), was diluted using distilled water and then spiked into Thrombocheck CaCl_2_ solution (Sysmex Corporation, Kobe, Japan) to achieve a final concentration of 4.1 µg/mL in the Thrombocheck CaCl_2_ solution. The CaCl_2_ solution containing r-tPA was prepared every 2 h to ensure reagent stability after spiking the tPA^2^. Each plasma sample (50 µL) was mixed with 50 µL of Thrombocheck APTT-SLA (Sysmex Corporation), including the activator and phospholipids, and incubated for 3 min at 37 ℃. Subsequently, the CaCl_2_ solution containing r-tPA was added, and the transmittance change was monitored at 660 nm. All measurements were conducted using an automated coagulation analyzer CS-5100 (Sysmex Corporation). An overview of the transmittance changes during the coagulation and fibrinolysis reactions is shown in Fig. 1b. For the transmittance change, the maximum and minimum values were defined as 0% and 100% in the clot-fibrinolysis waveform, respectively. The time taken to reach 50% of the transmittance at the midpoint between 0 and 100% on the curve was defined as the fibrinolysis time and expressed as CFWA-Lys (s)^19^. Thirty minutes was set as the maximum measurement time.

### GFC method

The plasma sample (120 µL) was mixed with the first reagent (120 µL), containing silica activator and 100 ng/mL tPA, and the mixture was incubated for 1 min at 37 ℃. The trigger reagent (60 µL) containing human thrombin and calcium was then added to the mixture, and the transmittance change was monitored at a wavelength of 940 nm using a Lysis Timer instrument^3^. Although the overview of the coagulation and fibrinolysis reactions is similar to that of CFWA (Fig. 1c), the coagulation phase is shorter because thrombin is used as the activator. The fibrinolysis time in GFC (GFC-Lys) is longer than CFWA-Lys, owing to the low tPA concentration. To calculate the fibrinolysis time, the first derivative curve indicating the velocity of the fibrinolysis reaction was obtained, and the fibrinolysis time was defined as the time taken to reach the point with the maximum value in the first derivative curve. One hundred and twenty minutes was set as the maximum measurement time.

### Sample preparation

Whole blood samples were collected in a vacuum blood collection tube containing a one-tenth volume of 0.105 mol/L (3.2%) trisodium citrate, and each sample was divided into four parts. Recombinant human tPA (HYPHEN BioMed) was spiked into each sample to achieve final concentrations of 0, 100, 500, and 1000 ng/mL in whole blood. These whole blood samples were used for ROTEM measurements, and the number of donors was 20 (age range: 23–46 years old; 60% female) and 20 (age range: 23–48 years old; 55% female) for EXTEM and INTEM, respectively; the number of samples with tPA concentrations of 0, 100, 500, and 1000 ng/mL was 20, 20, 20, and 17 for EXTEM and 19, 20, 17, and 18 for INTEM, respectively, owing to the lack of sample volume. After transferring the required volume of whole blood for ROTEM measurements to another aliquot, the remaining whole blood samples were centrifuged at 2000 × g for 15 min to obtain plasma samples. The number of plasma samples obtained was 39, 40, 37, and 35 for each tPA concentration, and these plasma samples were used for both the CFWA and GFC assays. Written informed consent was obtained from all donors. The study protocol was approved by the Institutional Review Board (approval number: 018-0062) and all experiments were performed in accordance with relevant guidelines and regulations.

### Data analysis

The distribution of fibrinolysis time in CFWA (CFWA-Lys) and GFC (GFC-Lys) was investigated at each tPA concentration for all samples, and statistical analysis was conducted between the tPA concentrations of 0 ng/mL and 100, 500, and 1000 ng/mL for each assay. Fibrinolysis time was expressed as a relative value which was calculated by dividing the fibrinolysis time by the median in the group of tPA concentration of 0 ng/mL. The relative values were also calculated for the LOT parameters of the EXTEM and INTEM data in the ROTEM method, and these relative values were compared among the three methods. The correlation between CFWA-Lys or GFC-Lys and the LOT parameters in EXTEM and INTEM of ROTEM was also investigated, along with the calculation of the correlation coefficients. The Mann–Whitney U test was used to compare the two groups. Statistical significance was set at *P* < 0.05. All statistical analyses were performed using JMP 17.1 software (SAS Institute Inc, Cary, NC, USA).

### Ethical approval

Written informed consent was obtained from all healthy donors. The study protocol was approved by the Review Board of Hokkaido University (approval number: 018-0062).

## Results

### Distribution of fibrinolysis time for CFWA-Lys and GFC-Lys assays at tPA concentrations of 0 to 1000 ng/mL

**Figure 2:**
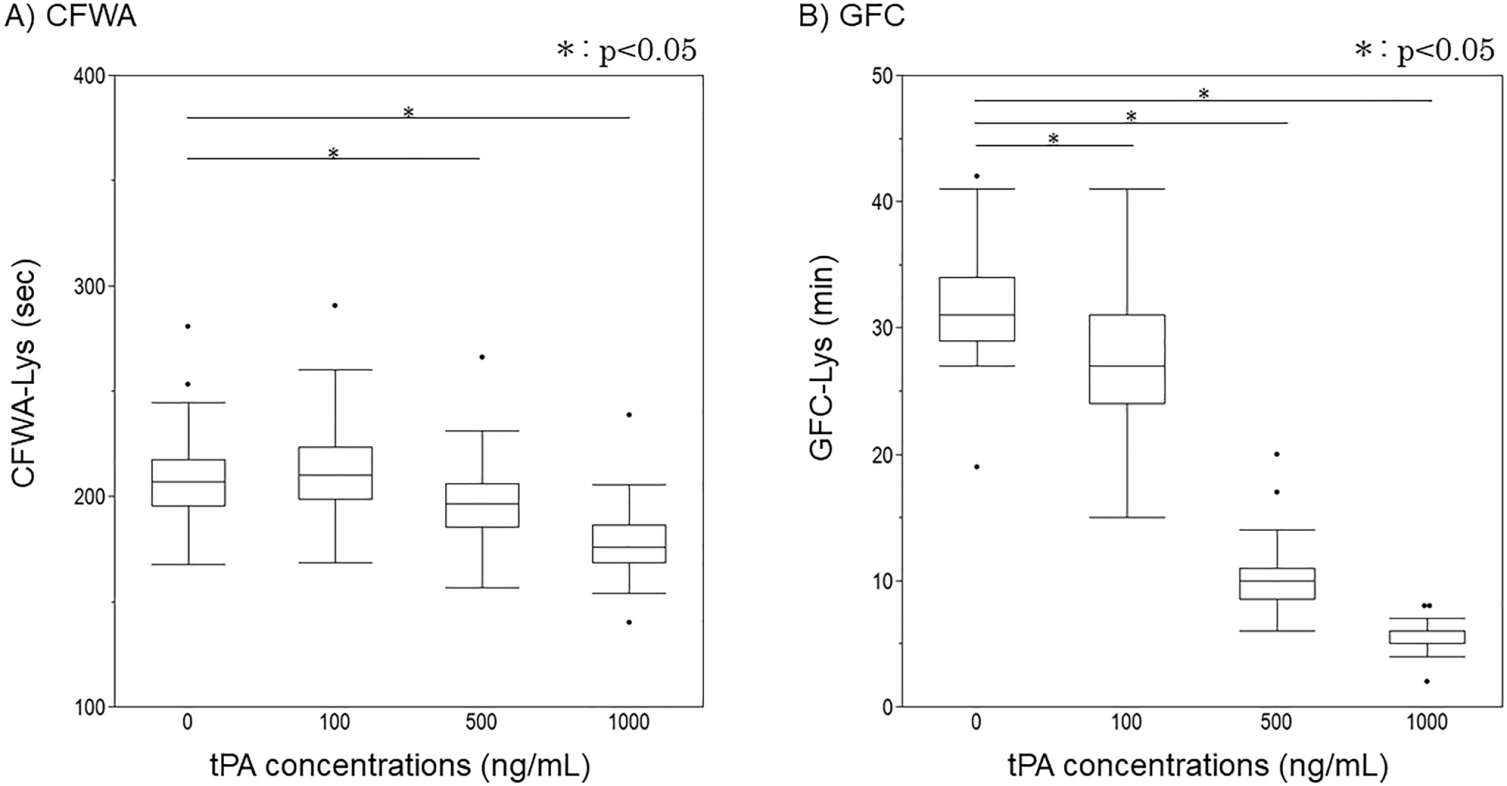
Relationship between the tPA concentrations and fibrinolysis time for CFWA and GFC assays. (**A**) CFWA, (**B**) GFC; *N.S.* indicates no significance. *indicates statistical significance (*P* < 0.05).

The distributions of CFWA-Lys and GFC-Lys were investigated at tPA concentrations of 0, 100, 500, and 1000 ng/mL (Fig. 2). For CFWA-Lys, statistical differences were found between the pairs of 0 and 500 ng/mL, and 0 and 1000 ng/mL. The median values of fibrinolysis times in the four concentration groups were 206.9, 210.0, 196.3, and 176.1 s, respectively. The range among these four values was only 33.9 s, indicating that the distributions overlapped among the four groups, although the differences were statistically significant. In contrast to CFWA-Lys, GFC-Lys showed no overlap in the distribution among tPA concentrations of 0 and 1000 ng/mL. Remarkable differences were observed in all pair comparisons. The median fibrinolysis time decreased from 31 to 6 min as the tPA concentration increased.

### Comparison of the relative values of the fibrinolysis time among the three methods

**Figure 3:**
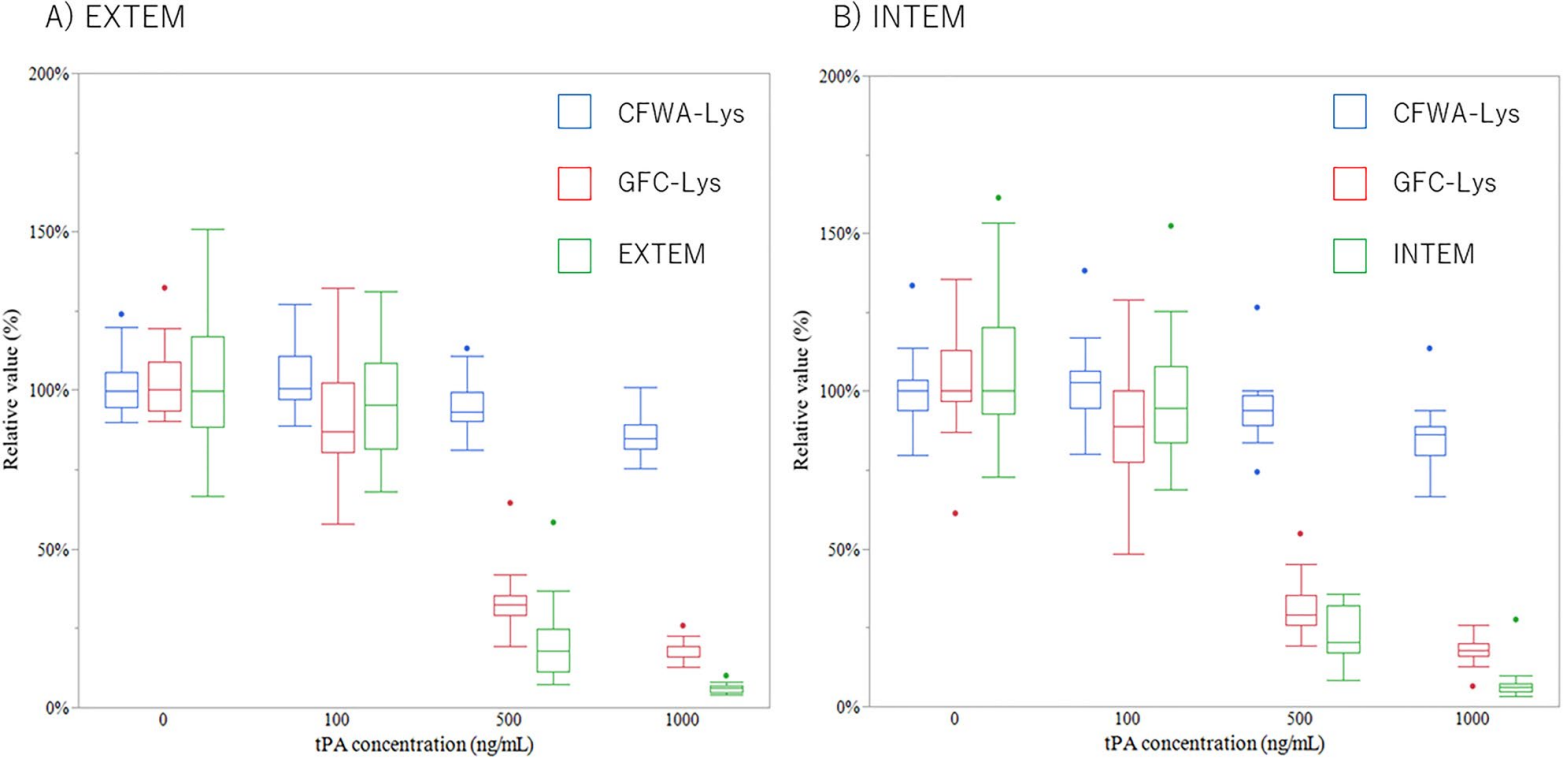
Relationship between the tPA concentrations and relative values of the fibrinolysis time among the three methods. The three methods were compared for each tPA concentration, and the relative values were used for unit normalization. (**A**) EXTEM (green) was compared with CFWA-Lys (blue) and GFC-Lys (red). (**B**) INTEM (green) was compared with CFWA-Lys (blue) and GFC-Lys (red). The statistical differences are recognized between 0 and 500/1000 ng/mL for CFWA-Lys, 0 and 100/500/1000 ng/mL for GFC-Lys, and 0 and 500/1000 ng/mL for ROTEM in both the EXTEM and INTEM graphs, respectively. All *P*-values are < 0.05.

The relative values of CFWA-Lys, GFC-Lys, and LOT in ROTEM were evaluated in each tPA-spiked concentration to compare these three assays in the same dimension (Fig. 3). The values were significantly reduced as tPA concentrations increased from 0 to 1000 ng/mL for GFC-Lys and both EXTEM and INTEM in ROTEM. In contrast to GFC-Lys and ROTEM, CFWA-Lys showed small difference among samples with different tPA concentrations.

### Correlations of CFWA-Lys and GFC-Lys with the ROTEM parameters

**Figure 4:**
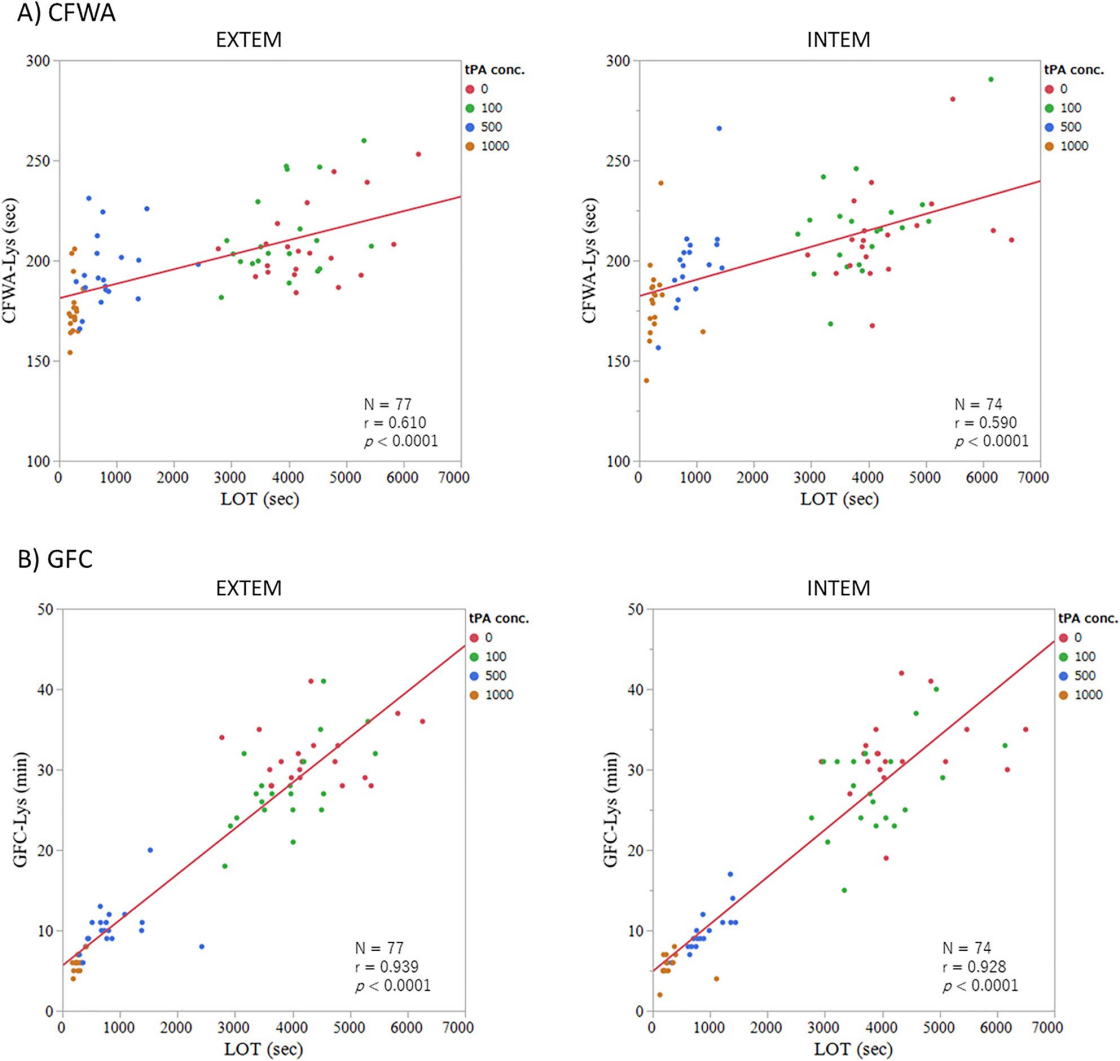
Correlations of CFWA-Lys and GFC-Lys with ROTEM parameters. The correlations with EXTEM and INTEM are shown for (**A**) CFWA and (**B**) GFC. The unit of tPA concentration is “ng/mL”.

The correlations with LOT in EXTEM and INTEM were investigated, and the correlation coefficients were 0.610 and 0.590 for CFWA-Lys and 0.939 and 0.928 for GFC-Lys, respectively (Fig. 4). The *P*-values were < 0.0001 for all correlations.

## Discussion

In this study, we compared the CFWA and GFC, which are global assays for coagulation and fibrinolysis, with ROTEM, which is the classical viscoelastic test for evaluating the fibrinolysis status, as the reference method. On comparing the GFC and ROTEM, it was found that the fibrinolysis time decreased as the tPA concentration increased, and the correlation coefficients between these two methods were more than 0.928. In contrast, the difference in CFWA-Lys between 0 and 1000 ng/mL tPA concentrations was less than those of GFC and ROTEM, and the correlation coefficients with ROTEM were 0.610 less than those with GFC. These results indicate that the fibrinolysis time in the CFWA method was not significantly affected by the tPA concentrations in the samples because the amount of tPA added at the time of measurement was 41 folds higher than that of the GFC assay. Accordingly, it was confirmed that CFWA-Lys has low sensitivity to the tPA concentration in the samples, whereas GFC-Lys adequately reflects the tPA concentration not only in 500 and 1000 ng/mL tPA samples but also in 100 ng/mL tPA samples. These results indicated that GFC-Lys reflected tPA concentrations more sensitively than CFWA-Lys, and the GFC assay showed better agreement because there were no overlapping values according to the tPA concentrations. This difference could be attributed to the tPA concentration added at the time of measurement. Thus, the characteristics of the global fibrinolysis assays, such as the CFWA and GFC, were largely dependent on the tPA concentration of the samples.

ROTEM detects global clot formation and dissolution in real time using whole blood samples, and several parameters related to coagulation and fibrinolysis were obtained from the measurements. Although LOT was employed in this study, other parameters of LI30, LI45, and LI60, which reflect the relationship of the amplitude at 30, 45, or 60 min after CT, respectively, also exist. We also investigated the relationships among CFWA-Lys, GFC-Lys, and LI30/LI45/LI60. Although the *P*-values for the correlation were < 0.0001 for all parameters, the correlation coefficients were 0.428–0.451 and 0.884–0.901 for CFWA-Lys and GFC-Lys, respectively, and the LI30/45/60 values plateaued at high tPA concentrations (data not shown). Therefore, we did not employ LI30/45/60 calculated from the amplitude parameters, and instead used LOT related to the fibrinolysis time.

It is known that tPA promotes hyperfibrinolysis by converting plasminogen to plasmin. In patients in critical care, its blood concentration is elevated and a hyperfibrinolytic status is frequently recognized^19–21.^ The physiological tPA concentration in healthy individuals is 720 pg/mL^22^. The final tPA concentrations in CFWA and GFC were approximately 1900 and 140 times higher than those in healthy individuals, respectively, indicating that the concentrations were higher than those under physiological conditions. A large amount of tPA is released in patients with out-of-hospital cardiac arrest, the most severe ischemia–reperfusion injury in humans, where its concentration may be up to 250 times that in healthy individuals^23^. Therefore, because of the large amount of tPA used in the CFWA assay, CFWA has lower sensitivity to tPA concentrations in clinical samples than GFC. Patients with out-of-hospital cardiac arrest with high tPA concentrations showed a hyperfibrinolysis status and poor prognosis^23^. Measurements of tPA concentrations are required in these cases, and GFC would be useful.

Although CFWA demonstrated low sensitivity to tPA concentration in the samples, the measurements were conducted using an automated blood coagulation analyzer with other routine coagulation tests, including prothrombin time (PT), APTT, and other fibrinolysis markers, and the results were obtained within 30 min. Since the results include not only the fibrinolysis time but also other fibrinolysis markers, such as plasminogen and a_2_-plasmin inhibitor, they are useful for data interpretation and understanding the patient’s status. As GFC can accurately reflect the tPA concentration, this assay was considered useful for estimating the tPA concentration. However, the measurement time was longer than that of CFWA, with a maximum of 2 h. The coagulation and fibrinolysis situation may change significantly over time, especially in acute care patients, and the results may no longer be useful^24^. A specific Lysis Timer instrument was also required for the measurements. In ROTEM, the assay is conducted using whole blood samples obtained from a specific point-of-care device. The parameters related to the coagulation and fibrinolysis status can then be obtained from the reaction containing the existing blood cells. Especially, platelets are included in the assay. Platelets are activated in the coagulation reaction by thrombin, and fibrin is formed around the activated platelets and blood cells. Therefore, these data should be interpreted including the effects of these blood cells. The cellular components play an important role in the reaction in ROTEM. In contrast, plasma was used for CFWA and GFC, and the discrepancy in the correlation between ROTEM and the other two assays might be derived from the presence of blood cells. Furthermore, there are several differences between the CFWA and GFC assays. The triggers of the coagulation reaction were ellagic acid for the intrinsic pathway and silica/thrombin for the intrinsic and common pathways, respectively. This difference might be related to the thrombin generated in each reaction. The final sample volumes were 50/150 µL and 120/300 µL, respectively, resulting in different final fibrinogen concentrations in the reaction, which could affect clot formation and fibrinolysis reaction. Although the major cause of the difference in fibrinolysis times would be derived from tPA concentration in the assays, these differences might be related to the results. Understanding these characteristics is important for optimal data interpretation, and the most applicable assay should be selected based on the purpose.

### Limitations

This study has some limitations. The comparisons among the assays were conducted using only tPA-spiked samples from healthy donors. Clinical samples with high tPA concentrations from critical care patients should also be obtained, and it should be confirmed that there are no differences between the spiked and clinical samples with various coagulation and fibrinolysis situations. These coagulation and fibrinolysis factors could affect the results of the global fibrinolysis assays, and this tendency might vary depending on the patient’s background.

## Conclusion

The correlations of CFWA and GFC with ROTEM were statistically significant. These assays have the potential to reflect hyperfibrinolysis status with high tPA concentrations. The GFC assay proved to have better agreement with ROTEM compared with the CFWA assay. There are several differences between the CFWA and GFC assays, including the tPA concentration and measurement instruments, and it is important to understand these characteristics for selecting the most appropriate method based on the purpose.

## Data Availability

The corresponding author will disclose data when requested.
